# Socio-economic inequalities in minimum dietary diversity among Bangladeshi children aged 6–23 months: a decomposition analysis

**DOI:** 10.1038/s41598-022-26305-9

**Published:** 2022-12-15

**Authors:** Satyajit Kundu, Pranta Das, Md. Ashfikur Rahman, Md. Hasan Al Banna, Kaniz Fatema, Md. Akhtarul Islam, Shobhit Srivastava, T. Muhammad, Rakhi Dey, Ahmed Hossain

**Affiliations:** 1 Global Health Institute, North South University, Dhaka, 1229 Bangladesh; 2 School of Public Health, Southeast University, Nanjing, 210096 China; 3 Faculty of Nutrition and Food Science, Patuakhali Science and Technology University, Patuakhali, 8602 Bangladesh; 4 Department of Statistics, University of Nebraska–Lincoln, Lincoln, NE 68583-0963 USA; 5 Department of Statistics, University of Dhaka, Dhaka, 1000 Bangladesh; 6 Development Studies Discipline, Khulna University, Khulna, 9208 Bangladesh; 7 Department of Food Microbiology, Faculty of Nutrition and Food Science, Patuakhali Science and Technology University, Patuakhali, 8602 Bangladesh; 8 Statistics Discipline, Science Engineering & Technology School, Khulna University, Khulna, 9208 Bangladesh; 9 Department of Survey Research and Data Analytics, International Institute for Population Sciences, Mumbai, 400088 India; 10 Department of Family & Generations, International Institute for Population Sciences, Mumbai, 400088 India; 11 Department of Statistics, Government Brajalal College, National University of Bangladesh, Gazipur, 1704 Bangladesh; 12 College of Health Sciences, University of Sharjah, 27272 Sharjah, United Arab Emirates; 13 Department of Public Health, North South University, Dhaka, 1229 Bangladesh

**Keywords:** Nutrition, Paediatrics, Public health

## Abstract

This study aimed to measure the socio-economic inequalities in having minimum dietary diversity (MDD) among Bangladeshi children aged 6–23 months as well as to determine the factors that potentially contribute to the inequity. The Bangladesh Demographic and Health Survey (BDHS) 2017–2018 data were used in this study. A sample of 2405 (weighted) children aged 6–23 months was included. The overall weighted prevalence of MDD was 37.47%. The concentration index (CIX) value for inequalities in MDD due to wealth status was positive and the concentration curve lay below the line of equality (CIX: 0.1211, p < 0.001), where 49.47% inequality was contributed by wealth status, 25.06% contributed by the education level of mother, and 20.41% contributed by the number of ante-natal care (ANC) visits. Similarly, the CIX value due to the education level of mothers was also positive and the concentration curve lay below the line of equality (CIX: 0.1341, p < 0.001), where 52.68% inequality was contributed by the education level of mother, 18.07% contributed by wealth status, and 14.69% contributed by the number of ANC visits. MDD was higher among higher socioeconomic status (SES) groups. Appropriate intervention design should prioritize minimizing socioeconomic inequities in MDD, especially targeting the contributing factors of these inequities.

## Introduction

Malnutrition among children and infants is a leading public health dilemma that causes half of all death of children each year, which may occur due to a lack of diversity, quantity, and quality of food intake^1–3^. Children with malnutrition are at greater risk of childhood diseases like diarrheal illnesses and contaminations, reduced adult height, and mental and physical growth hindrance, which may hamper educational accomplishment, consequently leading to minimal economic efficiency^4–6^. In several countries, nearly one-fourth of these children are not receiving the balanced nutrition they require to grow appropriately, specifically in the first 1000 days^3,7^. Balanced nutrition through optimum infant and young child feeding (IYCF) practices^2^, particularly in the early stages of life, ensure growth and increases the children's survival rate^8^ by reducing the risk of several chronic and lifestyle-related diseases^9–11^. Additionally, appropriate IYCF practices are significantly related to children's social, educational, and cognitive growth and improvement^9,12^. According to World Health Organization (WHO) and the United Nations Children’s Fund (UNICEF), minimum dietary diversity includes eight food groups, from which a child needs to take at least five food groups to receive the optimal level of food required for their overall growth^13^. Increasing dietary diversity is essential for sufficient intake of vital nutrients and ensuring a high-quality diet^14,15^, which is the key to evaluating nutritional sufficiency among young children and infants^16^. Thus, to ensure enough nutrition, prevent malnutrition, and promote healthy growth and development from infancy to adulthood, a diet from diversified food groups is essential^15,17^.

Even though dietary diversity is well recognized as an essential component, only 29 percent of children aged 6–23 months get the facility of the criteria of dietary diversity around the world^18^. Due to this problem, approximately149 million (22%), 45 million (7%), and 39 million (6%) under-five children are affected by stunting, wasting, and overweight, respectively, across the world^19,20^. In Bangladesh, among children aged under five years old, 31% are stunted, 8% are wasted, 2% are overweighted, and 22% are underweighted, respectively^21^. Although the fourth Health, Population, and Nutrition Sector Program (HPNSP) aims to decline stunting from 36 to 25% by 2022, the rate is still not yet achieved and remains unacceptable^21^. Nguyen et al., argued that socioeconomic status has a crucial impact in ensuring MDD and the households with richest quintiles has more access to and ability to afford the foods from diversified groups than the lowest quintiles^22^. While a previous study conducted in rural Bangladesh found a causal association between low dietary diversity and child stunting^23^. Understanding the socioeconomic disparities in having MDD might be crucial for improving child health and well-being given the high rates of malnutrition, stunting, underweight, and wasting in Bangladesh^24^. Hence, knowing the reasons behind inadequate diet and socioeconomic inequalities in MDD is a possible way to find solutions to mitigate these problems.

The MDD in South Asia varies across countries^25^, while MDD in Bangladesh is increased from 23.8% in 2011 to 28.8% in 2018^24^. The MDD and, eventually, child nutrition has been found to be closely correlated with a number of factors in previous research. According to previous studies in Bangladesh, dietary diversity is associated with age of the child, maternal age, family income, and place of residence^17^. Another study by Sarah and Tina found that household wealth status, maternal education, antenatal care visit, and exposure to media were significant determinants of MDD among Bangladeshi children^24^.

Although some studies have estimated the influencing factors of children's minimum dietary diversity utilizing Demographic and Health Survey (DHS) data^24,26,27^, there exists little information on socioeconomic inequalities in minimum dietary diversity among Bangladeshi children aged 6–23 months. Even, as per our knowledge, no research in Bangladesh has been conducted to examine whether socioeconomic inequalities in MDD among children exists or not. However, understanding inequalities in MDD is needed in order to develop comprehensive intervention to improve child health and nutritional status. To enhance the understanding of the socioeconomic inequality gap, this study aimed to assess the prevalence of minimum dietary diversity among Bangladeshi children aged 6–23 months and evaluate the inequalities and relative significance of socioeconomic resources. Our focus was to create evidence and a baseline by identifying the contributing factors of socioeconomic inequalities in MDD among Bangladeshi children so that the policymakers and public health experts can design appropriate intervention focusing on the most contributing factors of inequalities to enhance the children’s dietary diversity and hence the overall child health.

## Methods

### Data source and sampling method

The study used the data of 2017–2018 Bangladesh Demographic and Health Survey. The survey was conducted under the National Institute of Population Research and Training (NIPORT), Research and Development operational plan of 4th Health, Population and Nutrition Sector Program (HPNSP), and Health education and Family welfare Division of the Ministry of Health and Family Welfare under Training. The sampling frame used in the survey is the list of enumeration areas (EAs) of the 2011 Population and Housing Census of the People’s Republic of Bangladesh which was given by the Bangladesh Bureau of Statistics (BBS). The two-stage stratification of households were done in which in the first stage using probability proportional to EA size 675 EAs (425 from rural areas and 250 from urban areas) were selected. To obtain sampling frame for second stage a complete household listing was carried out in the selected EAs. In the second stage, on an average 30 households were selected in every EAs to provide an estimate of all the demographic and health indicators. Then all the eligible women aged 15–49 years were interviewed to obtain the data. More information regarding the sampling methodology can be found in 2017–2018 BDHS report^21^. Data regarding child nutritional status were asked to the ever married women having children.

### Outcome variable

Minimum dietary diversity is the outcome variable of the study. The variable was coded as “Yes” if the child aged 6–23 months received food from at least 5 groups out of 8 groups of food in the previous day of the survey otherwise the variable was coded as “No”^21^. The eight food groups are (1) Breastfeeding (2) Grains, roots and tubers (3) Legumes and nuts (4) Dairy products (milk, yogurt, cheese) (5) Flesh foods (meat, fish, poultry and liver/organ meats) (6) Eggs (7) Fruits and vegetables rich with vitamin A (8) Other fruits and vegetables.

### Explanatory variables

Household wealth status (Poorest, poorer, middle, higher, highest) and mother education level (No education, primary, secondary, higher) are considered as the main explanatory variables as these variables were considered to calculate the concentration index of dietary diversity. Along with mother education level and wealth status, age group (15–19, 20–24, 25–29, 30–49), residence (urban, rural), partner education level (No education, primary, secondary, higher), currently working (Yes, No), post-natal visited (Yes, No), number of ANC visits (< 4 visits, ≥ 4 visits), sex of the child (Male, Female), number of living children (1, 2, ≥ 3 children), media exposure (Yes, No), and division (Barisal, Chittagong, Dhaka, Khulna, Mymensingh, Rajshahi, Rangpur, Sylhet) are also considered to conduct the decomposition of the observed concentration index. In addition, child’s age was also categorized into 6–12, 13–18, and 19–23 to observe the concentration in the age groups. BDHS calculated the wealth index based on several indicator variables. These indicators consisted of the variables relating household’s asset, characteristics, ownerships and utility services; such as materials of floor, materials of roof, availability of technological assets like television, mobile phones, and refrigerators, water source for drinking, cooking and washing, type of cooking fuel used, toilet facilities, and land ownership, livestock ownership, etc. Then principal component analysis was used on those indicator variables after standardizing the variables and wealth index was calculated using the calculated factor loadings. Then the wealth index was ordered and divided into five twenty percent sections. The sections are named as poorest, poorer, middle, richer, richest where lowest section was termed as poorest and the highest section was termed as richest^28^. The variable media exposure was coded as “Yes” if the woman reads newspaper/magazine at least once a week or watches television at least once a week or listens to radio at least once a week otherwise the variable was coded as “No”. The variable number of living children was categorized into 1, 2, and ≥ 3 children using the variable v218 in the child dataset. Number of ANC visits for the recent child of the respondent was categorized into < 4 visits and ≥ 4 visits using the variables m14 in the child dataset. The variable currently working and post-natal visited was coded using the variable v714 and m70 in the child dataset respectively. The rest of the variables are used as recorded by BDHS. All the variables in the study are selected based on the previous literatures addressing minimum dietary diversity or any Infant and Young Child Feeding (IYCF) practices^15,17,24,29,30^.

### Statistical analysis

**Figure 1:**
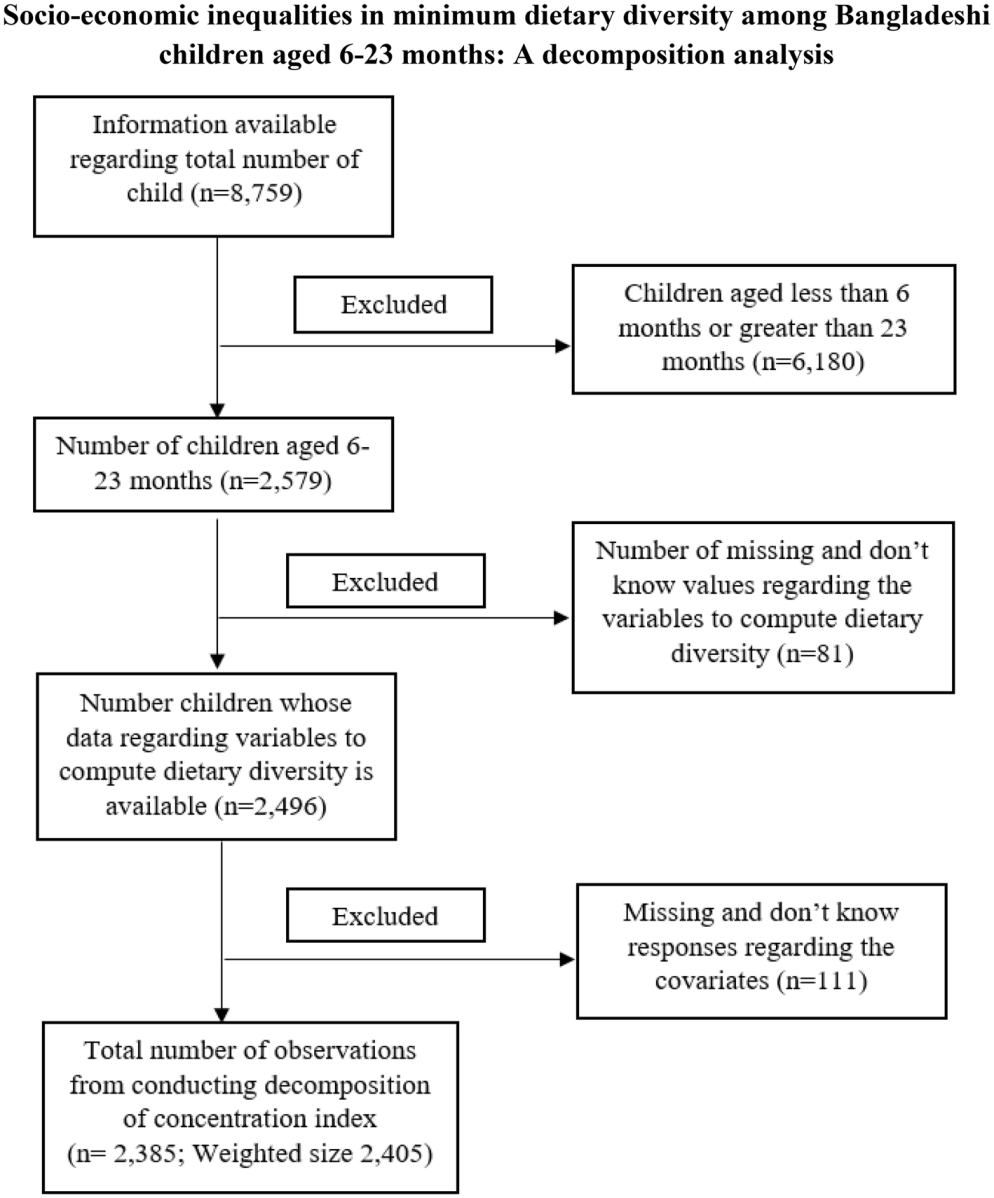
Flowchart of the how the used sample size ultimately reached.

Before conducting the analysis the raw data was filtered such as at the first stage data of 6–23 months aged child were filtered. The second sage filtration is that the missing and don’t know responses deletion regarding the variables used to compute dietary diversity. Then in the successive stages some other filtration were applied. At last data of size 2,385 children (unweighted) were obtained. The complete process of filtration and how many data deleted in each stage of filtration is depicted in Fig. 1. The characteristics of the data and the distribution of dietary diversity among the explanatory variables are tabulated and mapped.

To see whether there is an inequality in having minimum dietary diversity with respect to mother education level and wealth status, concentration curves were plotted. The concentration curve of minimum dietary diversity was also obtained with respect to mother’s education level and wealth status for each age group of the child. Concentrative curve with respect to wealth status shows the inequality in minimum dietary diversity by plotting the cumulative proportion of having minimum dietary diversity against the cumulative proportion of respondents regarding wealth status. A 45° line indicates the line of equality and any deviation from the line indicates the presence of inequality. If the concentration curve is below the line of equality it indicates that having minimum dietary diversity is concentrated among the respondents with higher wealth status and the concentration curve above the line of equality indicates that having minimum dietary diversity is concentrated among the respondents with lower wealth status. Similarly the concentrative curve with respect to mother education level is plotted and interpreted in similar way.

To quantify the level of concentration, concentration index (CIX) was calculated. The formula developed by Kakwani^31^, Jenkins^32^, and Kakwani et al.^33^ was used to calculate the CIX which is known as convenient covariance approach. The formula as follows-

$$CIX=\frac{2}{\mu }cov\left(h,r\right);$$

where $\mu$ is the weighted mean of the indicator which concetration to be caculated in this case it is the dietary diversity; r is the fractional rank of people in the distribution of variable by which concentration will be calculated (Here it is education level and wealth status); h represents the variable which concentration is to be calculated in this study it is media exposure; $cov(h,r)$ represents the covariance between h and r. CIX takes values within the closed interval of -1 and + 1. The closer the value of CIX to + 1 the higher the concentration in the upper quantile of the variable by which concentration is calculated and closer the value of CIX to -1 the higher the concentration in the lower quantile of the variable by which concentration is calculated.

Finally, to see the contribution of explanatory variables on the CIX, decomposition of CIX with respect to the explanatory variables were conducted. To do the decomposition the method developed by O’Donnell et al.^34^ was used. The method starts by fitting the following regression line-

$$y=\alpha +\sum_{k}{\beta }_{k}{X}_{k}+\varepsilon ;$$

where ${\beta }_{k}$ is the coefficient of kth explanatory variable, ${X}_{k}$ is the kth explanatory variable and $\varepsilon$ is the random error term.

Now using the results from above regression model the CIX for dietary diversity can be decomposed as

$$CI=\sum_{k}{(\beta }_{k}{\overline{X} }_{k}/\mu ){C}_{k}+{GC}_{\epsilon }/\mu ;$$

where ${\overline{X} }_{k}$ is the average of kth explanatory variable, ${C}_{k}$ is the concentration for kth explanatory variable, ${\beta }_{k}{\overline{X} }_{k}/\mu$ is the elasticity of dietary diversity with respect to kth explanatory variable, ${GC}_{\epsilon }/\mu$ represents the part of CIX that cannot be explained by the explanatory variables and all other notations hold their usual meaning. After performing the decompostion the percentage of contribution of each variables were plotted. Furthermore the concentration of the components of minimum dietary diversity was observed through calculating concentration curve and index with respect to wealth status and education level of the mothers. All the analysis were conducted using software R version 4.1.3 and Stata version 16. All the p-values where compared with 0.05 level of significance.

### Ethical approval

Secondary data set was used from the Demographic and Health Surveys (DHS) Programme for this study which is publicly available upon suitable request; therefore, further ethical approval was not required. Details of the ethical procedures followed by the DHS Program can be found in the BDHS report. All the procedures were performed in accordance with the relevant guidelines and regulations.

## Results

### Background characteristics

After the data cleaning data of 2,405 (weighted) children along with the characteristics of their parents are obtained. In the sample, 35.21% women belonged to age group 20–24 years, 48.86% women had secondary level of education, 74.01% resided in rural areas, and 55.80% were media exposed. Data contained information about 51.81% male children and 48.19% female children. There was 38.98% child belonging to age group 6–12 months, 34.47% belonging to 13–18 months age group, and rest 26.55% belonged to 19–23 months. Detailed presentation of the characteristics of the sample can be found in Table 1.

**Table 1:** Background characteristics of participants and distribution of minimum dietary diversity among the explanatory variables.

Variables	Categories	Total; N (%)	Having MDD; N (%)
**Overall prevalence**			37.47
Age group of children	6–12	938 (38.98)	234 (25.0)
	13–18	829 (34.47)	378 (45.57)
	19–23	638 (26.55)	288 (45.27)
Age group of mothers	15–19	461 (19.17)	163 (35.36)
	20–24	847 (35.21)	333 (39.32)
	25–29	597 (24.83)	220 (36.86)
	30–49	500 (20.79)	185 (37.00)
Education level of mother	No education	149 (6.19)	28 (18.79)
	Primary	650 (27.03)	189 (29.08)
	Secondary	1175 (48.86)	449 (38.21)
	Higher	431 (17.92)	235 (54.52)
Household wealth status	Poorest	497 (20.66)	129 (25.96)
	Poorer	519 (21.57)	181 (34.87)
	Middle	440 (18.29)	158 (35.91)
	Higher	488 (20.28)	192 (39.34)
	Highest	462 (19.20)	241 (52.16)
Partner education level	No education	326 (13.55)	88 (27.08)
	Primary	849 (35.29)	273 (32.16)
	Secondary	780 (32.42)	300 (38.41)
	Higher	451 (18.74)	241 (53.44)
Residence	Rural	1780 (74.01)	632 (35.51)
	Urban	625 (25.99)	269 (43.04)
Media exposure	No	1063 (44.20)	330 (31.04)
	Yes	1342 (55.80)	571 (42.55)
Mother currently working	No	1520 (63.20)	560 (36.84)
	Yes	885 (36.80)	342 (38.64)
Post-natal visited	No	835 (34.72)	286 (34.29)
	Yes	1570 (65.28)	615 (36.15)
Number of ANC visits	< 4 visits	1284 (53.39)	375 (29.21)
	≥ 4 visits	1121 (46.61)	526 (46.92)
Sex of the child	Male	1246 (51.81)	457 (36.68)
	Female	1159 (48.19)	444 (38.31)
Number of living children	1	943 (39.19)	387 (41.04)
	2	838 (34.83)	308 (36.75)
	≥ 3	625 (25.98)	207 (33.12)
Division	Barisal	140 (5.82)	
	Chittagong	487 (20.26)	
	Dhaka	615 (25.58)	
	Khulna	213 (8.86)	
	Mymensingh	206 (8.57)	
	Rajshahi	277 (11.52)	
	Rangpur	274 (11.40)	
	Sylhet	192 (7.98)	

### Prevalence of minimum dietary diversity

**Figure 2:**
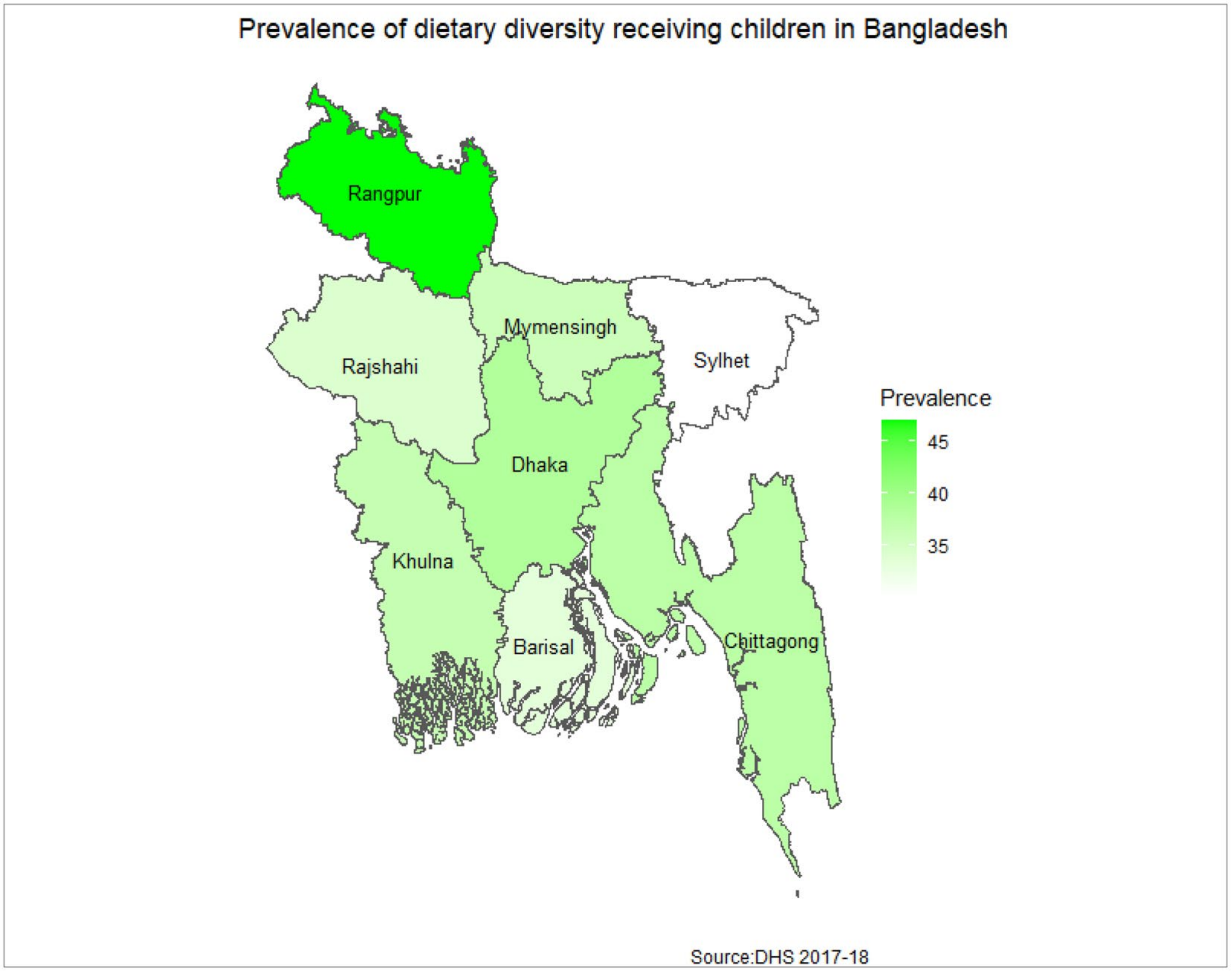
Distribution of minimum dietary diversity across eight divisions of Bangladesh. The map was created using R version 4.2.1 (https://cran.r-project.org).

The overall weighted prevalence of minimum dietary diversity was 37.47%. Dietary diversity of children is 39.32% prevalent with mother’s belonging to age group 20–24, 54.52% prevalent with mother’s having higher education level, and 52.16% prevalent with mother’s having wealth status (Table 1). From Fig. 2, it can be seen that minimum dietary diversity in Bangladeshi children was most prevalent in Rangpur division (47.08%) and least in Sylhet division (30.21%).

### Inequalities in minimum dietary diversity

**Figure 3:**
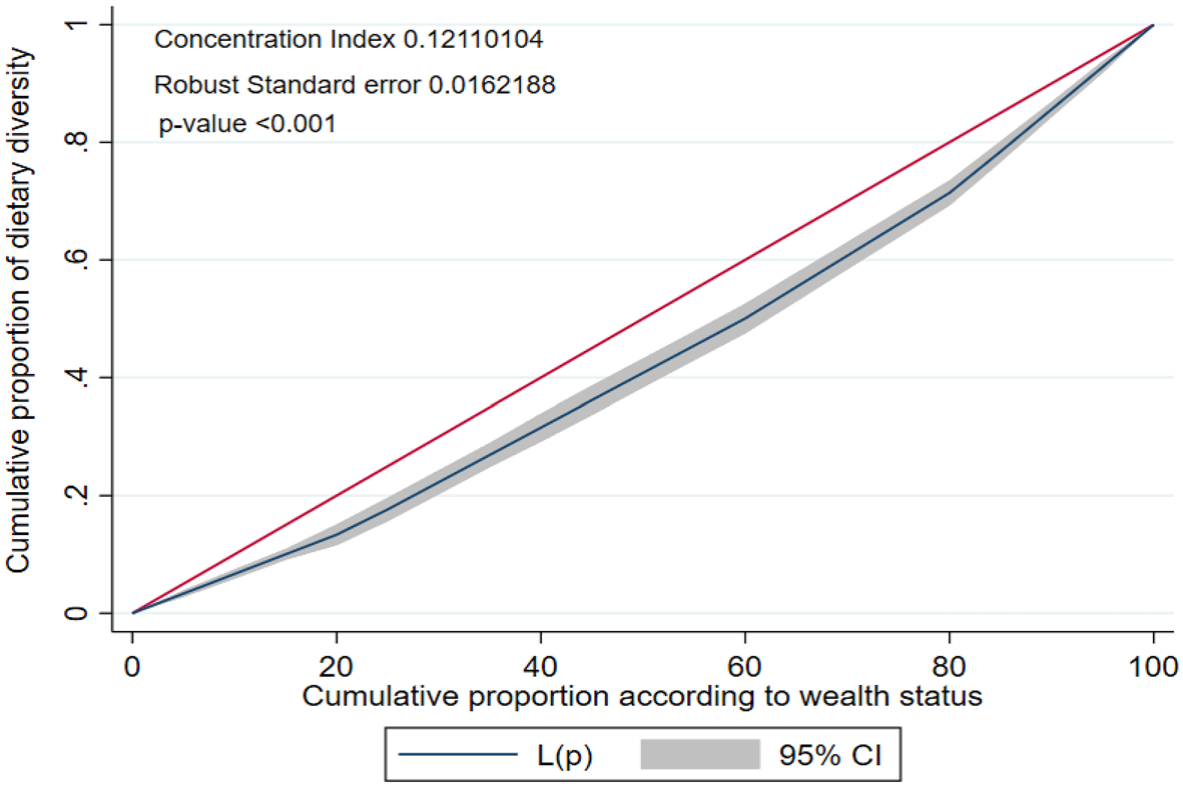
Concentration curve of dietary diversity against the wealth status.

**Figure 4:**
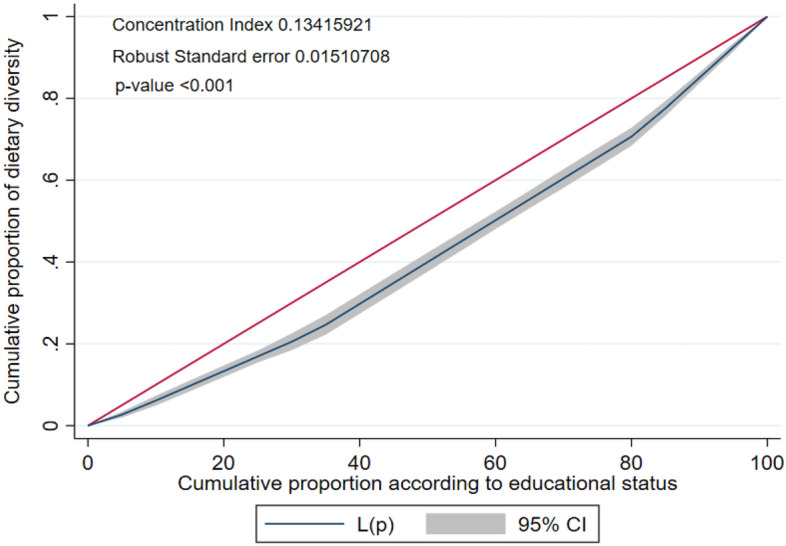
Concentration curve of dietary diversity against the education level.

From the concentration curve of dietary diversity against the wealth status it can be observed that the curve is below the 45° line so the minimum dietary diversity of children is more concentrated among the mother with higher wealth status (Fig. 3). The CIX regarding wealth status was 0.1211 and the p-value (< 0.05) indicates the significance of CIX. Therefore, minimum dietary diversity of children is significantly concentrated among the higher wealth status respondents. Similarly, from the Fig. 4 with the value of CIX and associated p-value, it can be concluded that minimum dietary diversity of children is significantly concentrated among the mother with upper education level.

### Inequalities in the components of minimum dietary

From the concentration curve of breastfeeding against the wealth status and education level of mothers, breasting is significantly concentration among the lower wealth status respondents whereas with the education level of the mothers it is not significantly concentrated. The foods groups dairy products, eggs, Vitamin A rich vegetables and fruits, other vegetables and fruits, legumes are significantly concentrated among higher wealth status and higher education level mothers. The food groups flesh and grains are not significantly concentrated with respect to wealth status but significantly concentrated with respect to education level (please see Supplementary Figs. S1–S16 online).

### Inequalities in minimum dietary diversity by age group

From the concentration curve of minimum dietary diversity with respect to both wealth and educational status of mother, we observed that MDD is concentrated among the children having mothers with higher educational and wealth status in each age group of the child. The value of CIX was highest in the 6–12 months child age group with respect to both educational and wealth status and lowest in 13–18 months age group for both educational and wealth status (please see Supplementary Figs. S17–S22 online).

### Decomposition of concentration index

The decomposition of CIX in respect to wealth status and education level were presented in Tables 2 and 3, respectively. Decomposition of CIX was done to see the contribution of different explanatory variables on inequalities. The column percentage of contribution in both the table represents the relative contribution of each explanatory variables on the overall CIX. A negative percentage of contribution indicates that the factor helps to decrease the concentration and a positive percentage of contribution indicates that the variable helps to increase the observed inequality. In the observed CIX due to wealth status which is 0.1211, 49.47% contributed by wealth status, 25.06% contributed by education level of mother, 20.41% contributed by the number of ANC visits, 11.07% contributed by media exposure, and 9.75% contributed by mother’s partner education level. Overall, those were the factor which contributed most and increased the observed inequality regarding wealth status. The contribution of post-natal visit is − 6.69% which indicating that post-natal visit helps to decrease the observed CIX. Furthermore, only 2.08% of the observed CIX cannot be explained by the explanatory variables.

**Table 2:** Decomposition of the concentration index obtained with respect to wealth index.

Variables	Elasticity	Concentration Index (CIX)	Contribution to overall CIX = 0.1211	
			Contribution	Percentage of contribution
**Age group of mothers**				
15–19 (ref.)				
20–24	0.0350	0.0352	0.0012	1.02
25–29	0.0181	− 0.0124	− 0.0002	− 0.19
30–49	0.0177	0.0279	0.0005	0.41
Total			0.0015	1.24
**Education level of mother**				
No education (ref.)				
Primary	0.0829	− 0.2753	− 0.0228	− 18.84
Secondary	0.2045	0.0362	0.0074	6.12
Higher	0.1093	0.4187	0.0457	37.78
Total			0.0303	25.06
**Household wealth status**				
Poorest (ref.)				
Poorer	0.0432	− 0.3714	− 0.0161	− 13.25
Middle	0.0279	0.0273	0.0008	0.63
Higher	0.0391	0.4131	0.0161	13.32
Highest	0.0731	0.8079	0.0591	48.77
Total			0.0599	49.47
**Partner education level**				
No education (ref.)				
Primary	0.0065	− 0.2098	− 0.0014	− 1.13
Secondary	0.0128	0.1319	0.0017	1.39
Higher	0.0265	0.4330	0.0115	9.49
Total			0.0118	9.75
**Residence**				
Rural (ref.)				
Urban	0.0084	0.3970	0.0033	2.75
Media exposure				
No (ref.)				
Yes	− 0.03654	− 0.3669	0.0134	11.07
**Mother currently working**				
No (ref.)				
Yes	0.0513	− 0.1770	− 0.0091	− 7.50
**Post-natal visited**				
No (ref.)				
Yes	0.1327	− 0.0610	− 0.0081	− 6.69
**Number of ANC visits**				
< 4 visits (ref.)				
≥ 4 visits	0.1210	0.2004	0.0242	20.01
**Sex of the child**				
Male (ref.)				
Female	0.0362	− 0.0059	− 0.0002	− 0.18
**Number of living children**				
1 (Ref.)				
2	− 0.0341	0.0263	− 0.0009	− 0.74
≥ 3	− 0.0097	− 0.1457	0.0014	1.16
Total			0.0005	0.42
**Division**				
Barisal (ref.)				
Chittagong	0.0118	0.0685	0.0008	0.67
Dhaka	− 0.0025	0.2778	− 0.0007	− 0.57
Khulna	− 0.0044	− 0.0041	0.00002	0.02
Mymensingh	0.0068	− 0.2348	− 0.0016	− 1.32
Rajshahi	− 0.0044	− 0.1066	0.0005	0.38
Rangpur	0.0299	− 0.2782	− 0.0083	− 6.87
Sylhet	− 0.0026	− 0.0973	0.0003	0.21
Total			− 0.00898	− 7.48
Explained CIX			0.11852	97.92
Residual CIX			0.00528	2.08

**Table 3:** Decomposition of the concentration index obtained with respect to educational status.

Variables	Elasticity	Concentration Index (CIX)	Contribution to overall CIX = 0.1341	
			Contribution	Percentage of contribution
**Age group of mothers**				
15–19 (ref.)				
20–24	0.0345	0.0877	0.0031	2.29
25–29	0.0181	− 0.0296	− 0.0005	− 0.40
30–49	0.0177	− .1256	− 0.0022	− 1.65
Total			0.0005	0.24
**Education level of mother**				
No education (ref.)				
Primary	0.0829	− 0.6059	− 0.0502	− 37.43
Secondary	0.2045	0.1528	0.0312	23.28
Higher	0.1093	0.8206	0.0897	66.83
Total			0.0715	52.68
**Household wealth Status**				
Poorest (ref.)				
Poorer	0.0432	− 0.1550	− 0.0067	− 4.99
Middle	0.0279	− 0.0073	− 0.0002	− 0.15
Higher	0.0391	0.1354	0.0053	3.94
Highest	0.0731	0.3536	0.0259	19.27
Total			0.0243	18.07
**Partner education level**				
No education (ref.)				
Primary	0.0065	− 0.2039	− 0.0013	− 0.99
Secondary	0.0128	0.0868	0.0011	0.83
Higher	0.0265	0.5536	0.0147	10.95
Total			0.0145	10.79
**Residence**				
Rural (ref.)				
Urban	0.0084	0.0698	0.0006	0.44
**Media exposure**				
No (ref.)				
Yes	− 0.03654	− 0.1970	0.0072	5.36
**Mother currently working**				
No (ref.)				
Yes	0.0513	− 0.0822	− 0.0042	− 3.14
**Post-natal visited**				
No (ref.)				
Yes	0.1327	− 0.0357	− 0.0047	− 3.54
**Number of ANC visits**				
< 4 visits (ref.)				
≥ 4 visits	0.1210	0.1630	0.0197	14.69
**Sex of the child**				
Male (ref.)				
Female	0.0362	0.0118	0.0004	0.32
**Number of living children**				
1 (ref.)				
2	− 0.0341	0.0031	− 0.0001	− 0.08
≥ 3	− 0.0097	− 0.2619	0.0025	1.88
Total			0.0024	1.80
**Division**				
Barisal (ref.)				
Chittagong	0.0118	0.05	0.0006	0.44
Dhaka	− 0.0025	0.0079	− 0.00002	− 0.015
Khulna	− 0.0044	0.0484	− 0.0002	− 0.16
Mymensingh	0.0068	− 0.1035	− 0.0007	− 0.53
Rajshahi	− 0.0044	0.0081	− 0.00004	− 0.03
Rangpur	0.0299	0.0556	0.0017	1.24
Sylhet	− 0.0026	− 0.2030	0.0005	0.39
Total			0.00184	1.34
Explained CIX			0.13404	99.05
Residual CIX			0.00012	0.95

**Figure 5:**
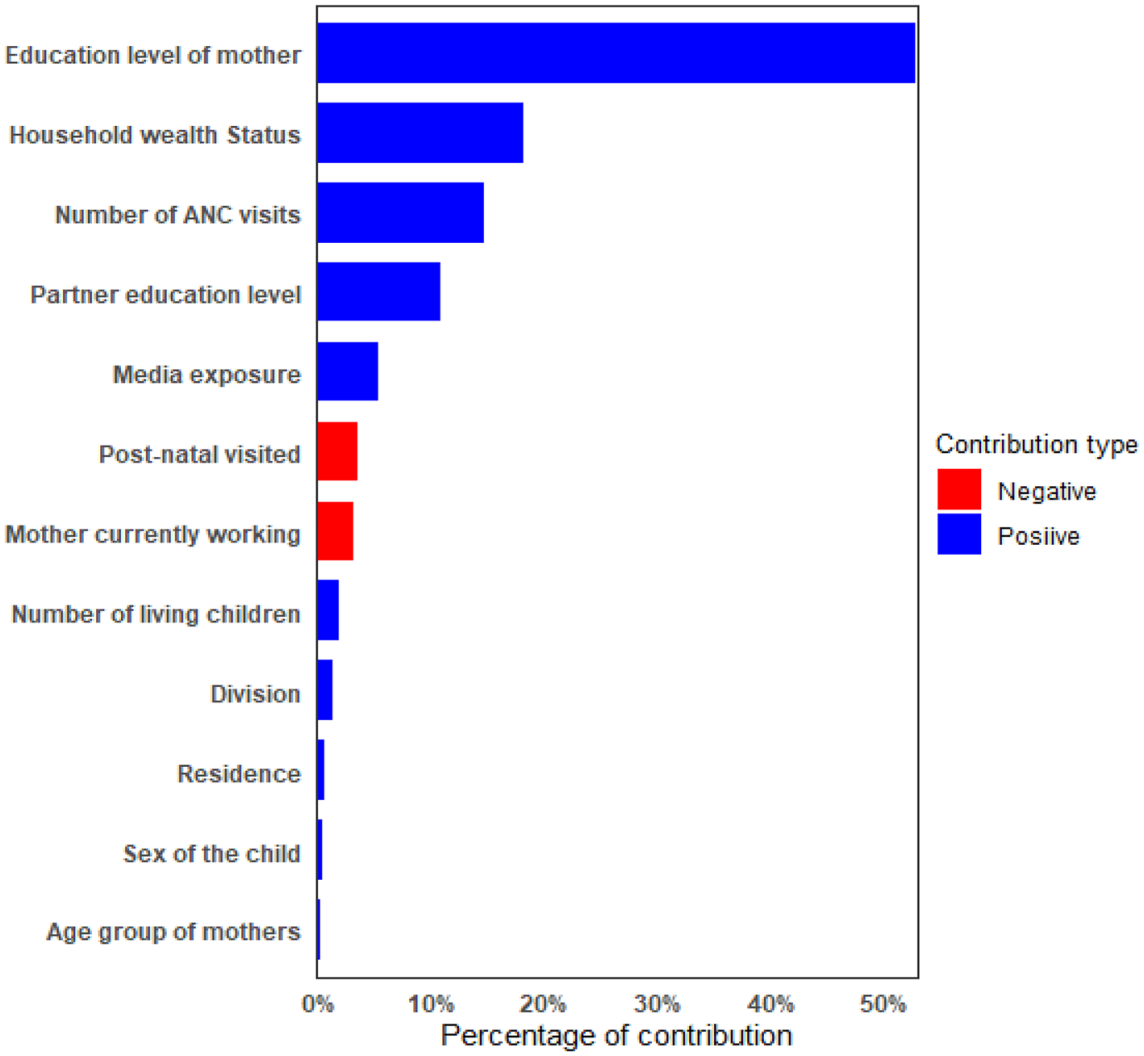
Percentage of contribution of variables on the Concentration Index of dietary diversity resulting from education level.

**Figure 6:**
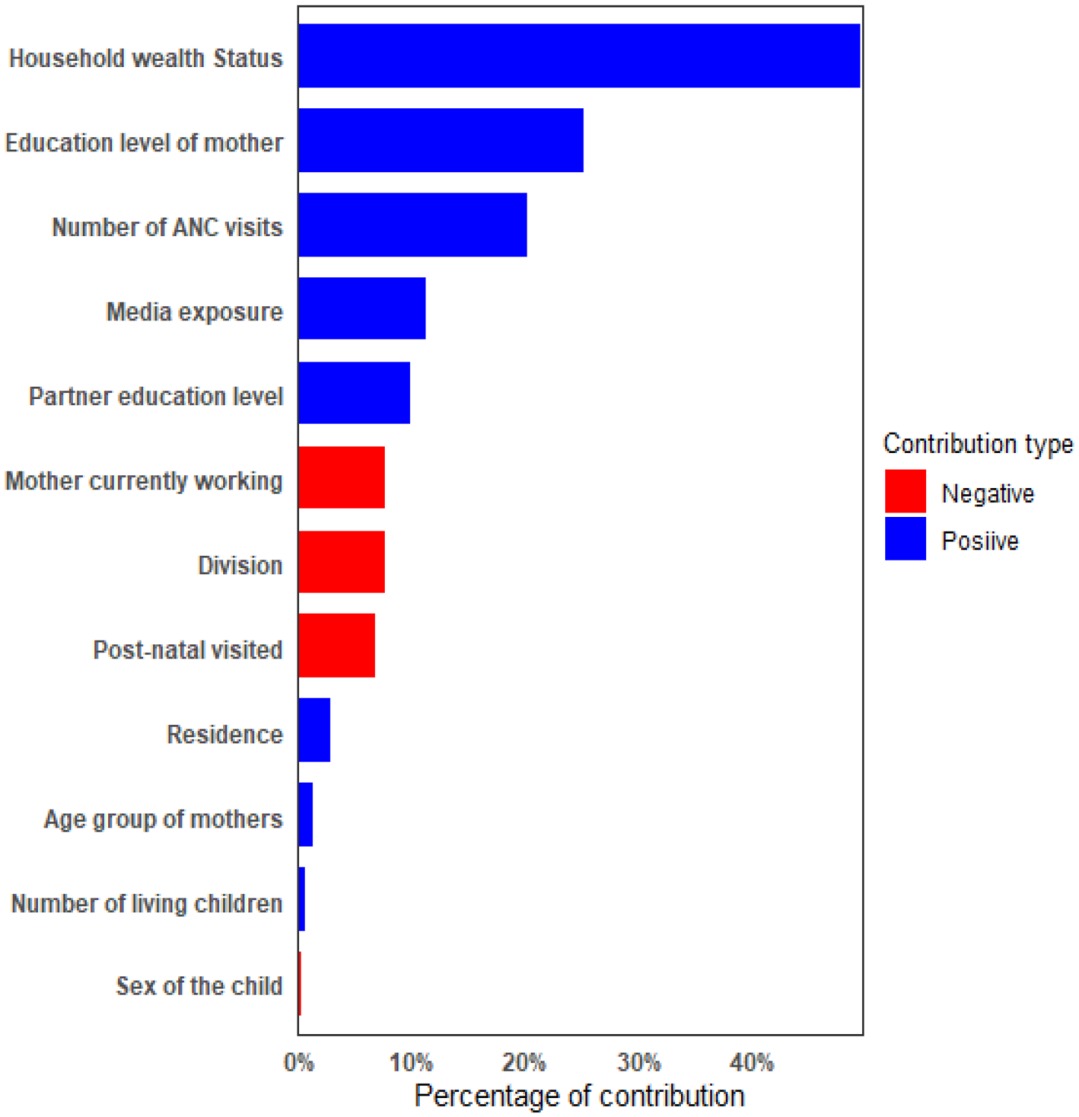
Percentage of contribution of variables on the Concentration Index of dietary diversity resulting from wealth status.

Similarly, the decomposition of CIX computed regarding education level of mother can be interpreted. From Table 3 it can be found that in the CIX = 0.1341, 52.68% contributed by education level of mother, 18.07% contributed by wealth status, 14.69% contributed by the variable number of ANC visits, 10.79% contributed by mother’s partner education level and 5.36% contributed by media exposure. These explanatory variables contributed most in the inequality and on its increase. Similar scenario can be observed regarding variable post-natal care visit. The percentage of contribution of the variables are presented in an order in the Figs. 5 and 6. Only 0.95% of this CIX could not be explained by the explanatory variables.

## Discussion

In the current study utilizing the large survey data from Bangladesh, we observed significant wealth-based and education-based inequality in children’s MDD. The factors such as parental (mothers’ and fathers’) education level, household wealth status, number of ANC visits and media exposure were found to be major contributors to the socioeconomic inequality in a child's MDD.

The results of our study are consistent with other national-level investigations conducted in other countries^35,36^, indicating a substantial relationship between the age of the child and dietary diversity. Older infants' readiness to take food in a variety of forms (such as tastes and textures) and their better acquaintance with food than younger infants could be two possible explanations for why they consume a more varied diet^37^. When looking at the inequalities by age groups of children with respect to both maternal education and household wealth status, higher disparities was observed among children from 6 to 12 months age group. This higher socioeconomic inequalities could be another explanation of why MDD was lower among children of lower age group; this finding may indicate the importance of education and socio-economic status in relation to meet the MDD among Bangladeshi children. Besides, further investigation is warranted to find out the in-depth reasons of higher inequalities in having MDD among children from lower age group in Bangladesh.

In this study, mothers’ and their partners’ education played a substantial role in the SES-related discrepancy for MDD among children. Educated caregivers may recognize the importance of food diversity in their children's development. This finding is consistent with the findings of other investigations^38,39^. A similar study in India found that maternal education was associated with consumption of essential food items and all food groups, and showed higher odds of adequately diversified dietary intake^40^.

The media as a source of information was also found to have a substantial contribution in SES-related inequalities in MDD. Media exposure has recently been suggested as an important predictor of women's empowerment, which may increase women's access to and control over resources^41^. Previous research has shown that the media can help people get better information and promote dietary diversity^42,43^. The circulation through mass media in many countries is shown to be a reliable source of delivering nutrition-related information and influence individuals’ behaviours^44,45^. In this regard, in the decomposition of contributing factors to education-based inequalities in receiving MDD, wealth status contributed only 18.07% whereas, in case of wealth-based inequalities, education contributed 25.06%. This suggests that education can play an important role than wealth in bringing positive outcomes related to MDD. Therefore, as reported in previous intervention studies^46^, counseling through post-natal care centres and media exposure may improve child dietary diversity even in areas with no food security activities.

The worst wealth status was linked to a low MDD in the current study, whereas the children from highest wealth quintile were observed to have a higher prevalence of MDD. This finding may be attributed to the resource availability where a wealthy family might have the financial capacity to buy a variety of items and feed their children a diversified diet. This is supported by previous evidence in other low- and middle-income countries where children from the poorest households being most at risk of not receiving the MDD^11,36,45,47,48^. Income has a good effect on children's intake of a variety food, according to other studies in India^40,49–51^. It is also evident from previous studies that people from lowest wealth quintile in Bangladesh do not utilize the health-care services if their children’s particular health problem is not severe because of not having proper financial security^52^.

Post**-**natal visits and ANC visits had a significant contribution to SES-related inequality in MDD. This may be because during these visits, mothers get nutrition awareness which helps in better MDD outcome^53,54^. Previous studies also suggested that access to ANC during pregnancy is indicative of meeting MDD^25^. Similarly, maternal visits to ANC reflected maternal access to services related to health and nutrition and they might obtain information about appropriate child feeding through a counselling session during ANC^55^.

Furthermore, children from urban communities in Bangladesh were more likely to receive MDD. This might be due to the fact that poor nutritional knowledge and awareness, poor resources, lower education rate and limited income opportunity are available in rural areas. Therefore, awareness building interventions and income-generating activities among people in rural areas may enrich the nutritional status of the children^27^. However, rural region was found to be a protective factor for MDD in multiple previous studies in Bangladesh, which is attributed to the increased access to livestock or home-grown produce with relatively low inputs by rural mothers^24,26,56^. Thus, future studies are warranted that focus on factors influencing rural–urban gradient in attaining MDD among Bangladeshi children. Further, administrative regions were significant influencing factors of SES-based differences in MDD in this study, and this suggests the need for studies based on regional disparities in MDD among children in Bangladesh.

The important limitations of this study include the cross‐sectional design which limits the causal direction of observed factors and the use of 24‐h recall data for MDD. Although 24-h maternal recall is recommended by the WHO to assess child dietary intake^57^, the measurement is subject to response bias, including errors in recall and social desirability. Similarly, the dietary diversity also relates to the aspects of food security, affordability and accessibility which are not covered in this study. Moreover, because this was a secondary data analysis, we were limited in the measurements available for women empowerment and are not included in the analyses. Future studies could expand on these, and bring out the influence of maternal decision-making power and other aspects of woman empowerment on SES- and education-based differences in children’s MDD. Despite the abovementioned limitations, inclusion of a large number of factors in the multivariable analysis that strengthens the validity of the findings was a major strength of this study. Also, in addition to the SES-related inequality in MDD, the current study analyzed the education-based inequality in MDD which provides a clearer picture of the contribution of several factors to differences in MDD among Bangladeshi children. Finally, since the study utilized data of a large sample from a nationally representative household survey, the results can be generalized at the country level.

In conclusion, there was significant wealth- and education-based inequalities in MDD among Bangladeshi children aged 6–23 months. The significant contributors to such inequalities included parents’ education, household wealth status, mothers’ media exposure, ANC visits and residential status. The findings suggest that these can be possible targets of future interventions to improve MDD among Bangladeshi children, particularly among those who belong to economically poor households or born to less educated mothers.

## Supplementary Information

Supplementary Figures.

## Data Availability

This study utilized publicly available Demographic and Health Surveys Program dataset of Bangladesh. The dataset of BDHS 2017-18 is available upon request in the following website: http://dhsprogram.com/data/available-datasets.cfm. As a third-party user, we do not have permission to share the data publicly on any platform.

## Abbreviations

MDD: Minimum dietary diversity
SES: Socioeconomic status
BDHS: Bangladesh demographic and health survey
IYCF: Infant and young child feeding
NIPORT: National institute for population research and training
EAs: Enumeration areas
PSU: Primary sampling unit
CIX: Concentration Index
CI: Confidence interval
ANC: Antenatal care
