# Supplementary material for: Socio-economic inequalities in minimum dietary diversity among Bangladeshi children aged 6–23 months: a decomposition analysis
Source: Sci Rep. 2022 Dec 15;12:21712. doi: 10.1038/s41598-022-26305-9 (PMC9755277; doi:10.1038/s41598-022-26305-9)
Supplement: Supplementary file 1 — Supplementary Figures. [file 41598_2022_26305_MOESM1_ESM.docx]

**
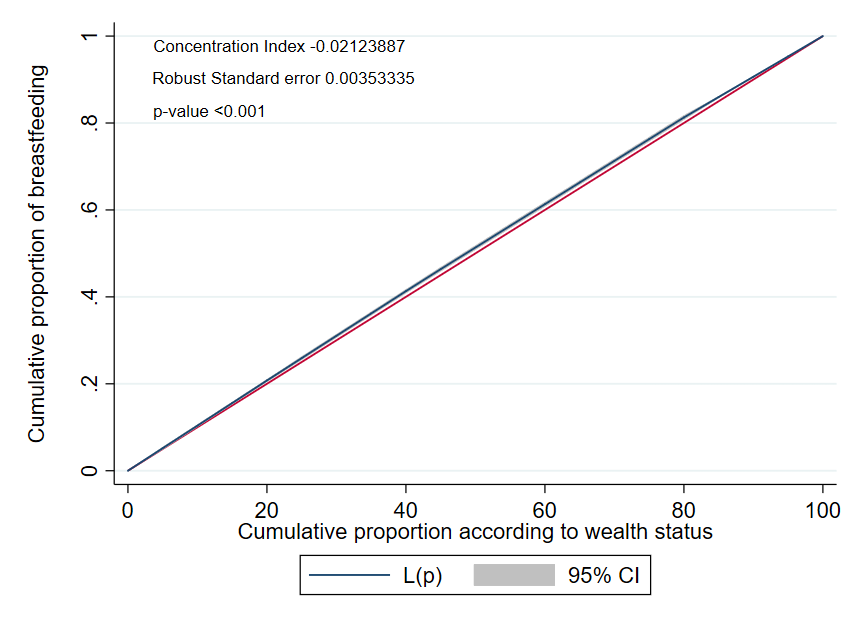
**

**Supplementary Fig. S1**. Concentration curve of breastfeeding against the wealth status


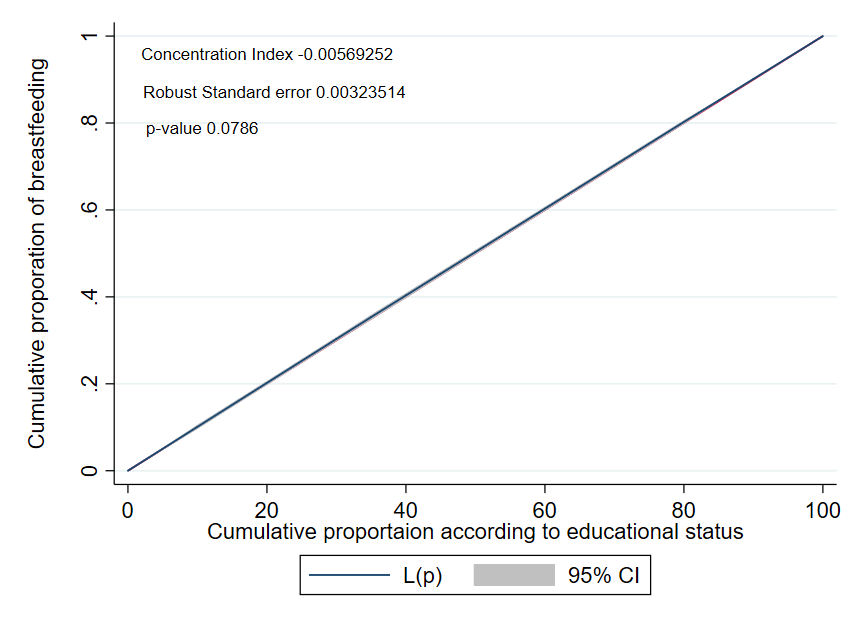


**Supplementary Fig. S2.** Concentration curve of breastfeeding against the education level


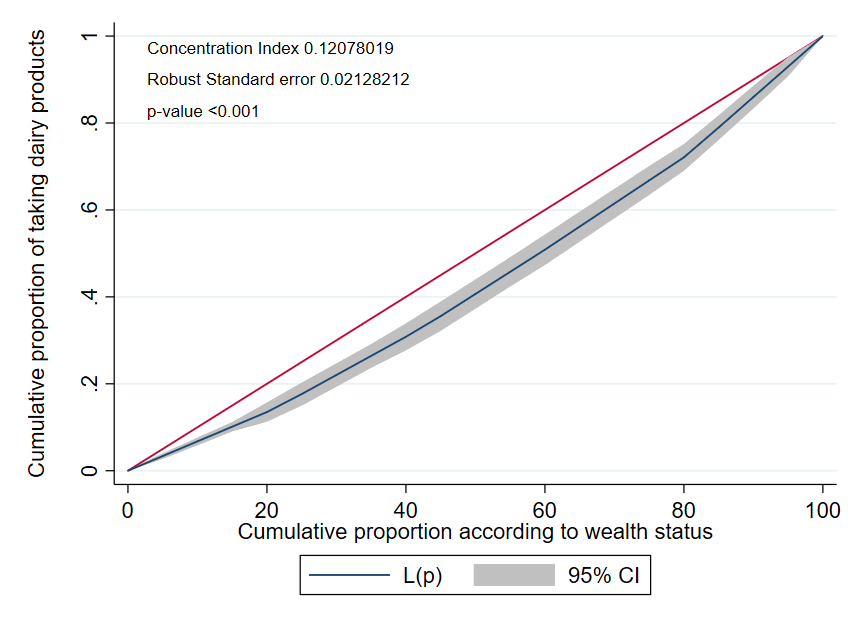


**Supplementary Fig. S3.** Concentration curve of dairy products against the wealth status

**
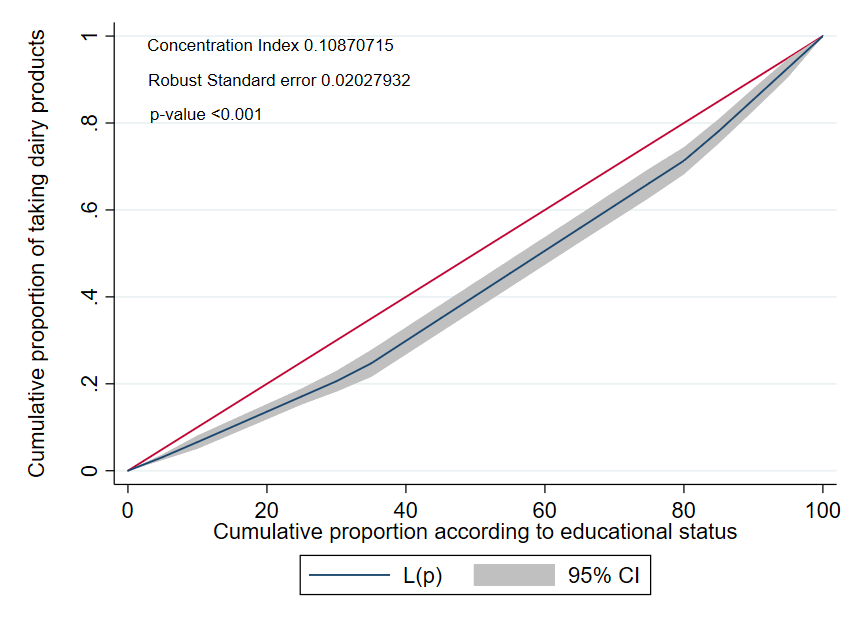
**

**Supplementary Fig. S4**. Concentration curve of dairy products against the education level

**
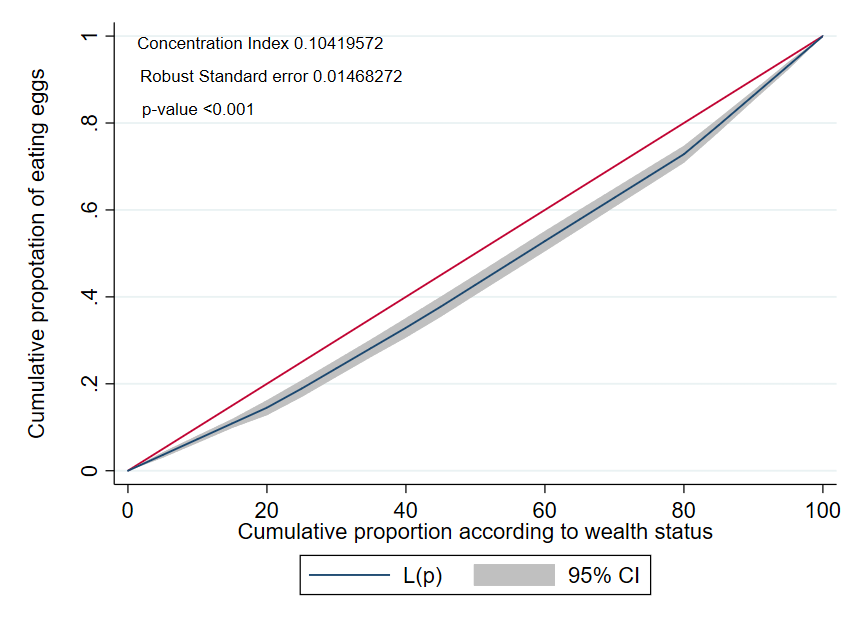
**

**Supplementary Fig. S5.** Concentration curve of eggs against the wealth status


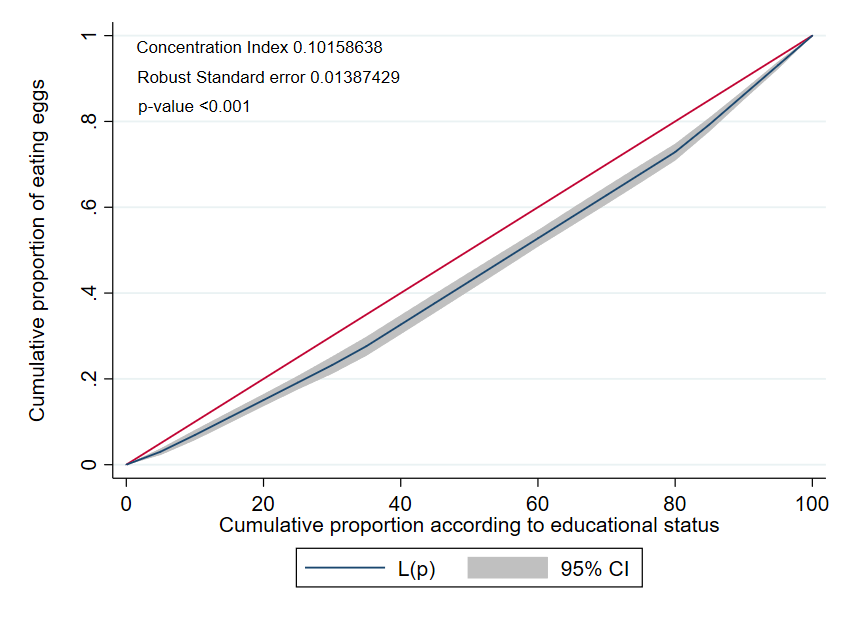


**Supplementary Fig. S6.** Concentration curve of eggs against the education level

**
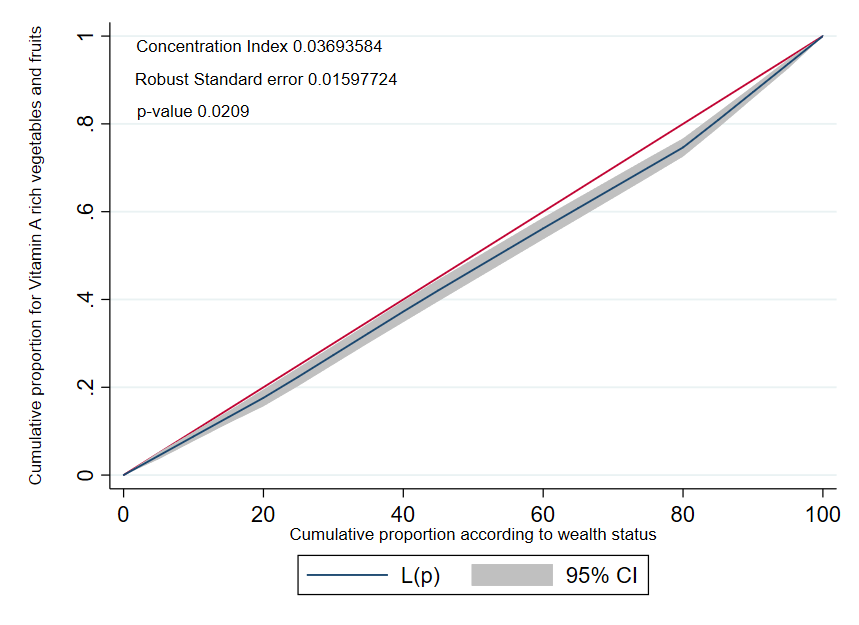
**

**Supplementary Fig. S7.** Concentration curve of Vitamin A rich vegetables and fruits against the wealth status


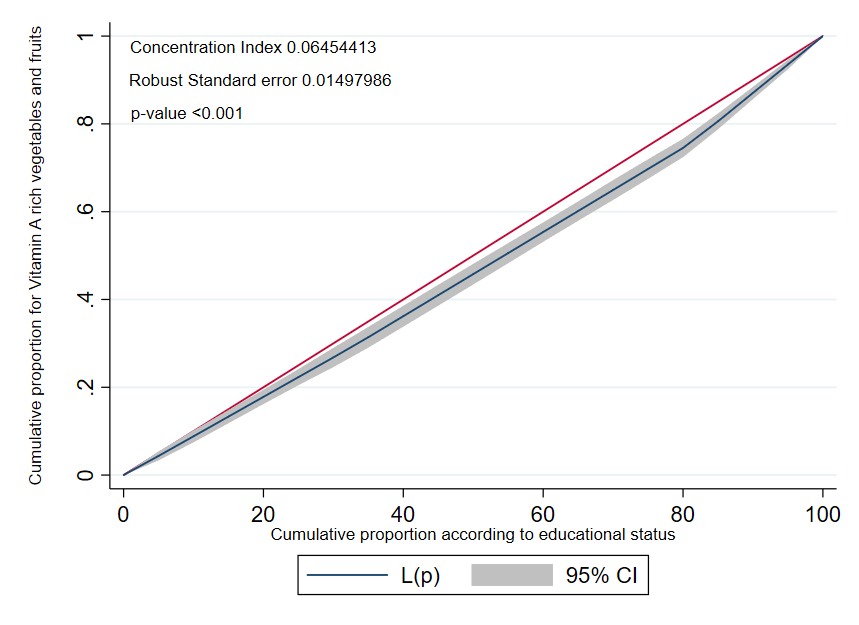


**Supplementary Fig. S8**. Concentration curve of Vitamin A rich vegetables and fruits against the education level


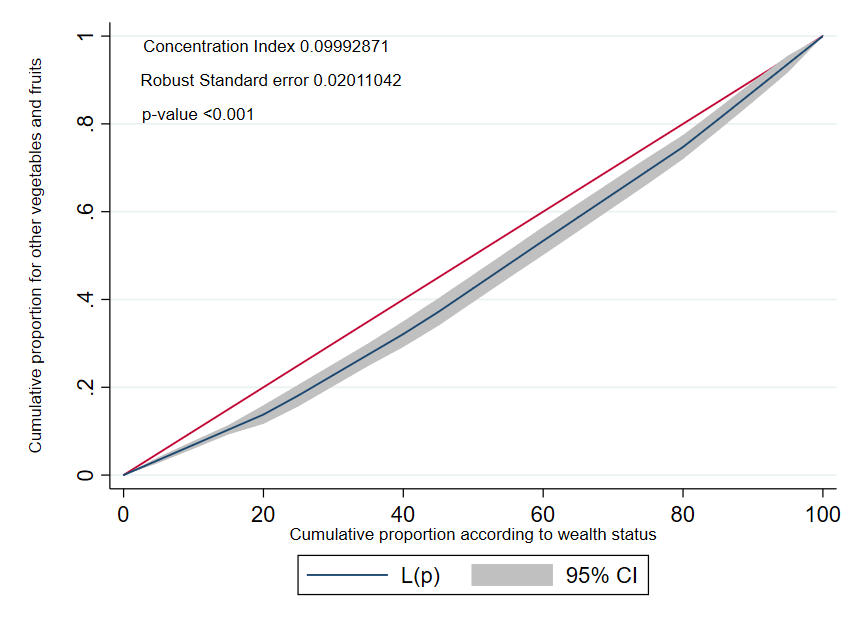


**Supplementary Fig. S9.** Concentration curve of other vegetables and fruits against the wealth status


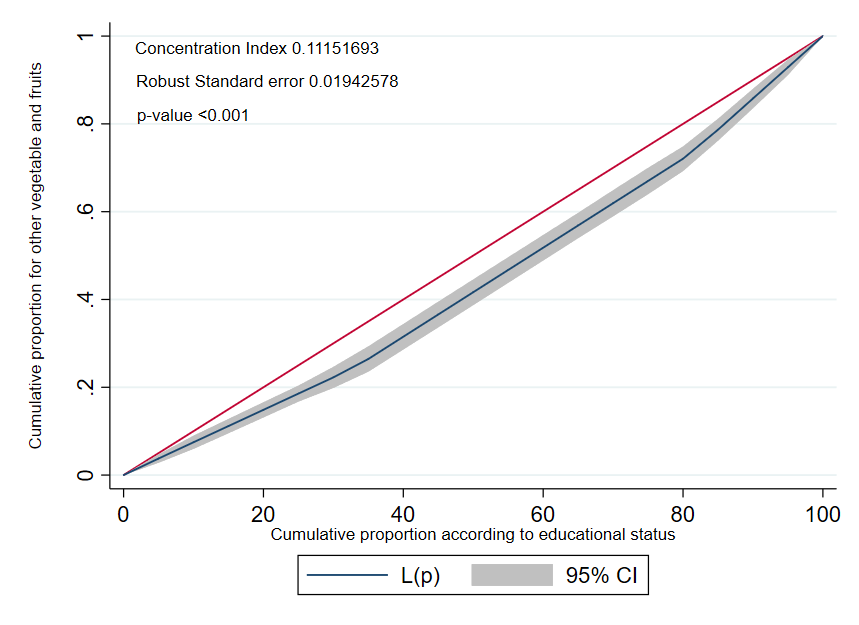


**Supplementary Fig. S10**. Concentration curve of other vegetables and fruits against the education level


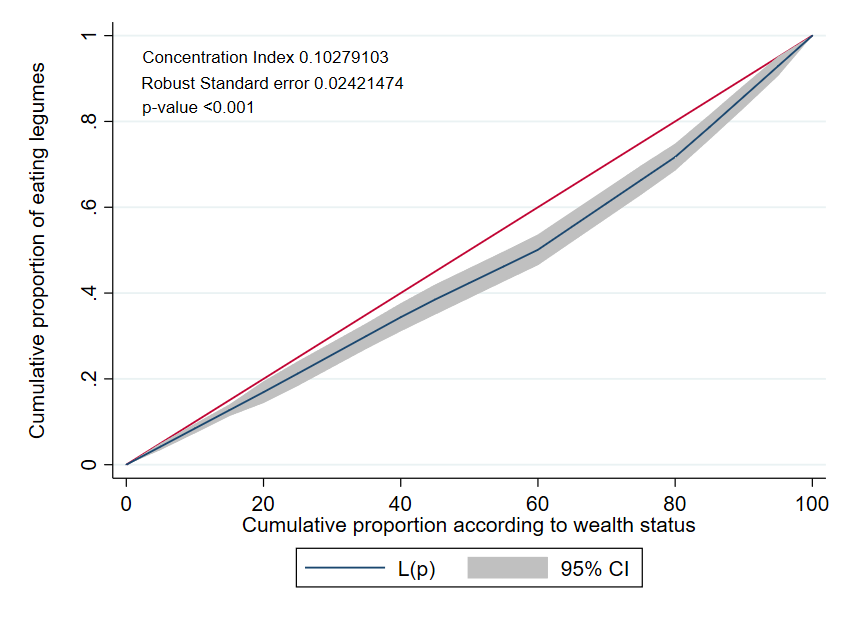


**Supplementary Fig. S11.** Concentration curve of eating legumes against the wealth status


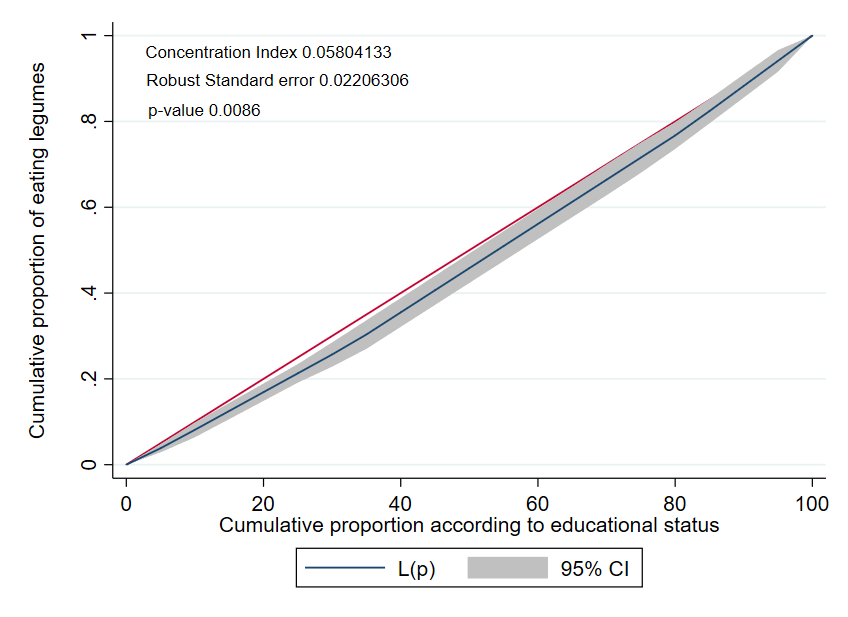


**Supplementary Fig. S12.** Concentration curve of eating legumes against the education level


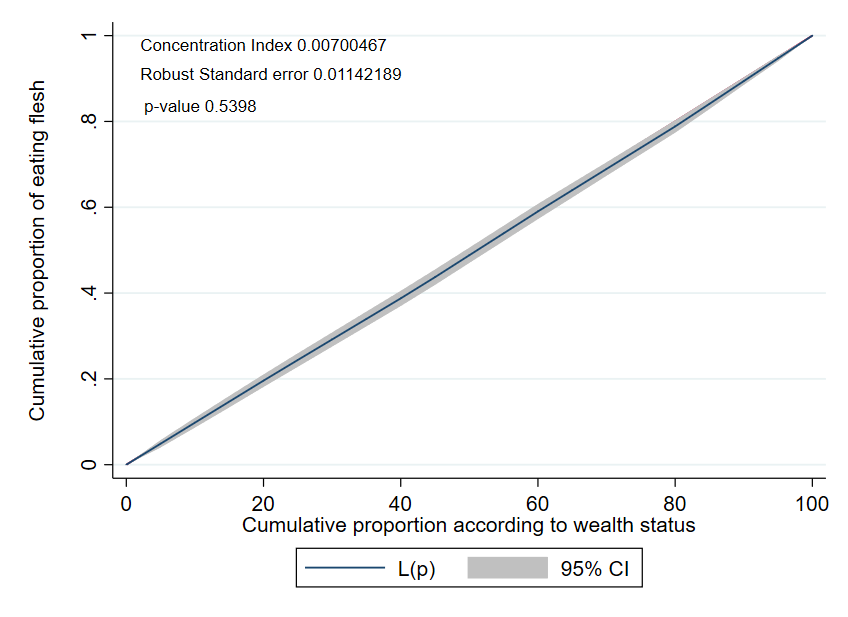


**Supplementary Fig. S13.** Concentration curve of eating flesh against the wealth status

**
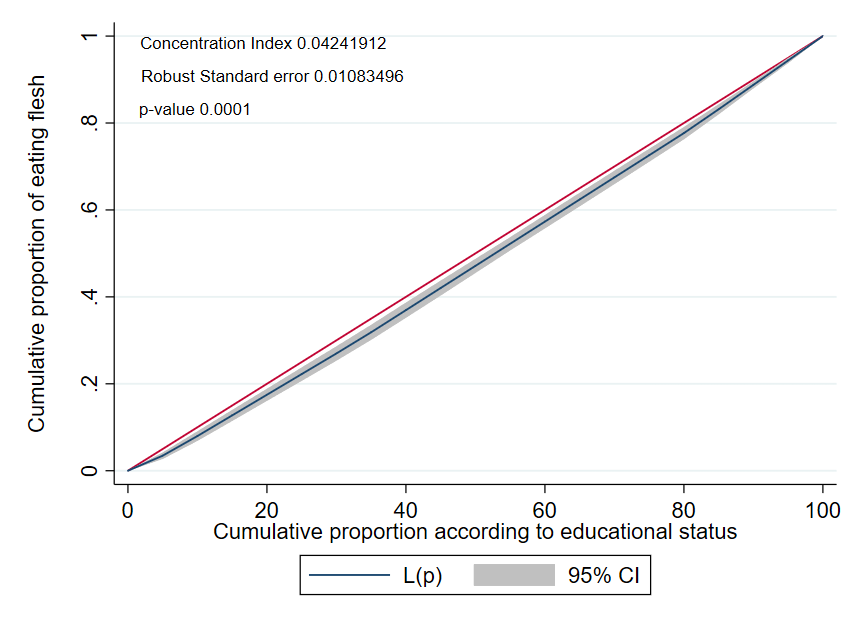
**

**Supplementary Fig. S14.** Concentration curve of eating flesh against the education level


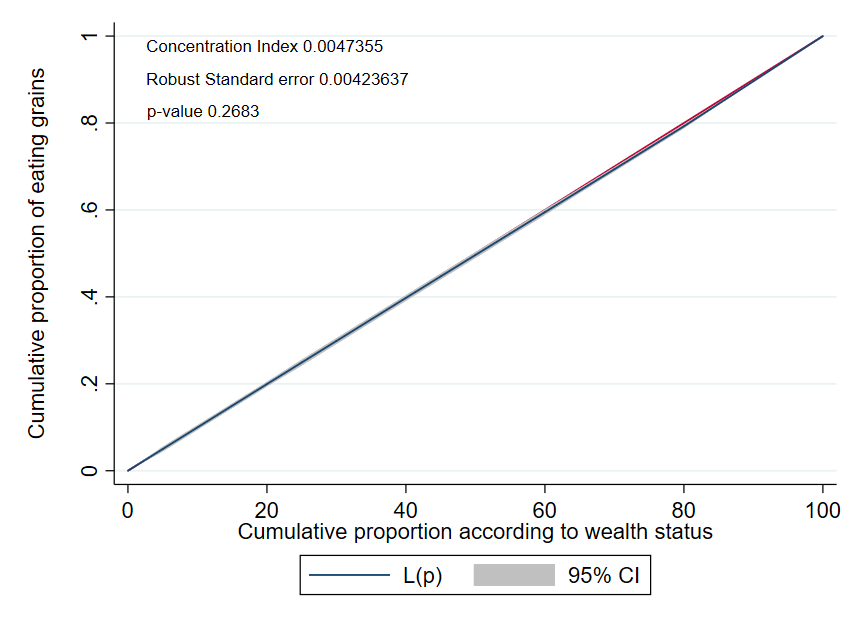


**Supplementary Fig. S15.** Concentration curve of eating grains against the wealth status


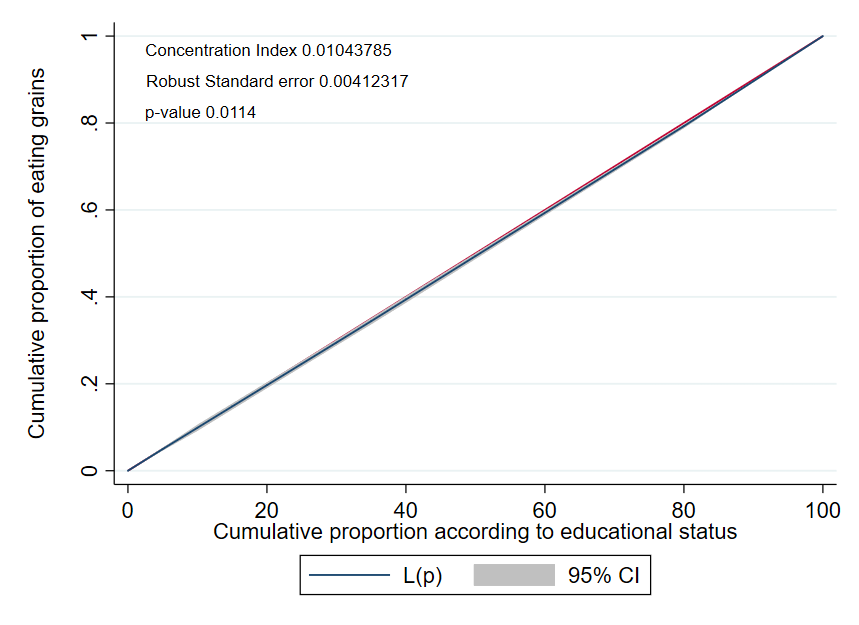


**Supplementary Fig. S16.** Concentration curve of eating grains against the education level


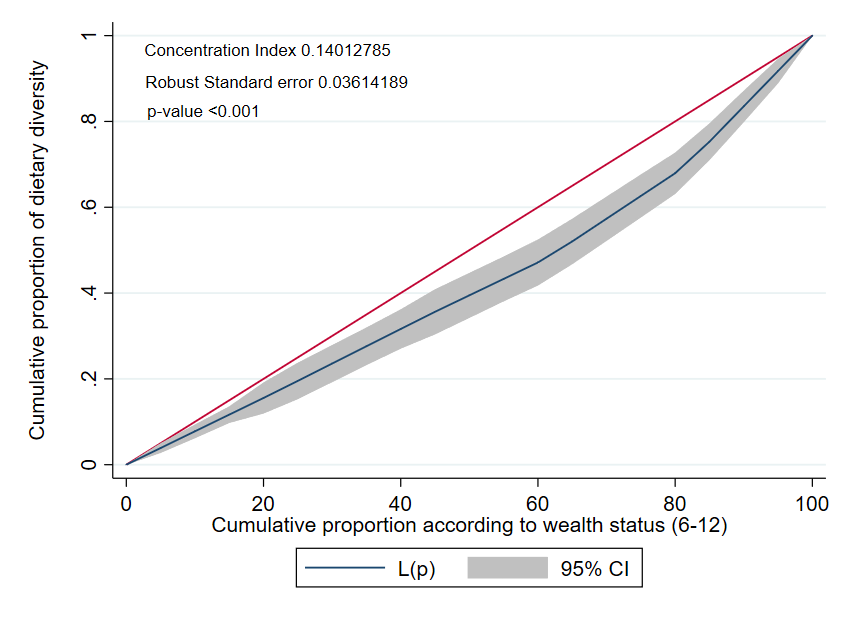


**Supplementary Fig. S17.** Concentration curve of dietary diversity among children aged 6-12 months against the wealth status of family.


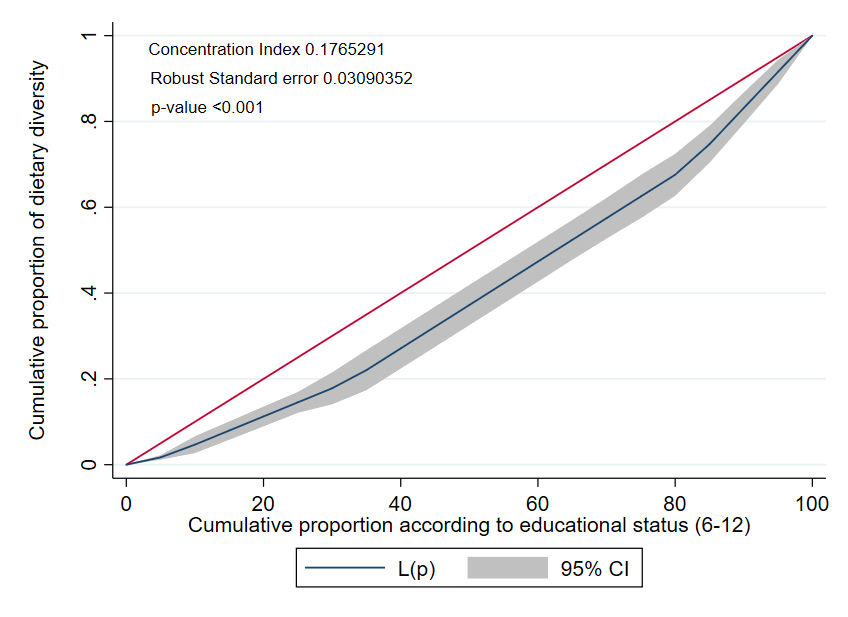


**Supplementary Fig. S18.** Concentration curve of dietary diversity among children aged 6-12 months against the educational status of family.


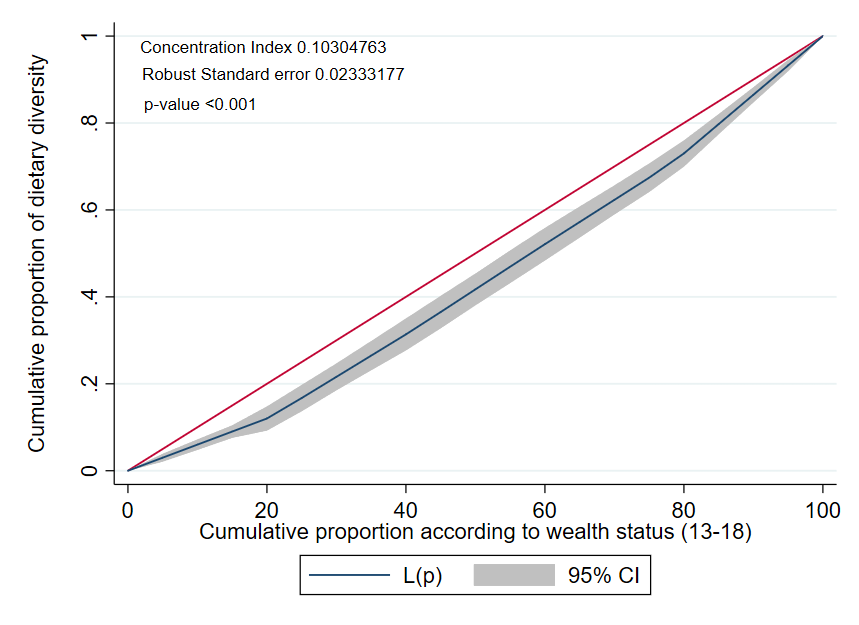


**Supplementary Fig. S19.** Concentration curve of dietary diversity among children aged 6-12 months against the wealth status of family.


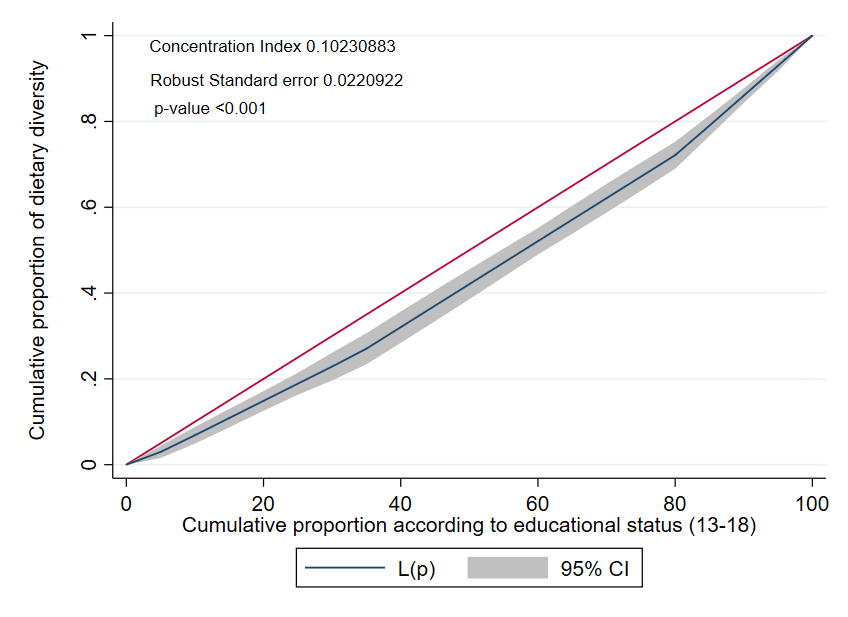


**Supplementary Fig. S20.** Concentration curve of dietary diversity among children aged 6-12 months against the educational status of family.


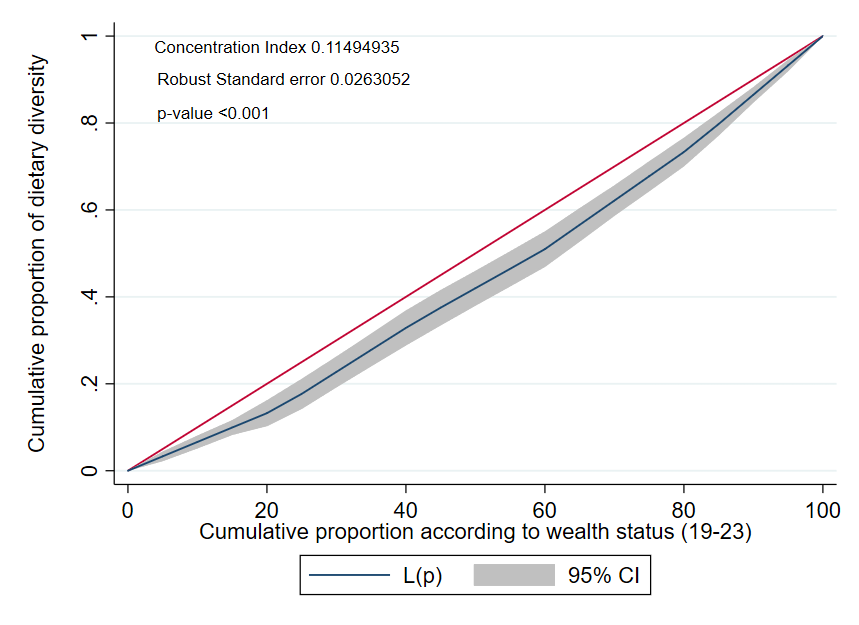


**Supplementary Fig. S21.** Concentration curve of dietary diversity among children aged 6-12 months against the wealth status of family.


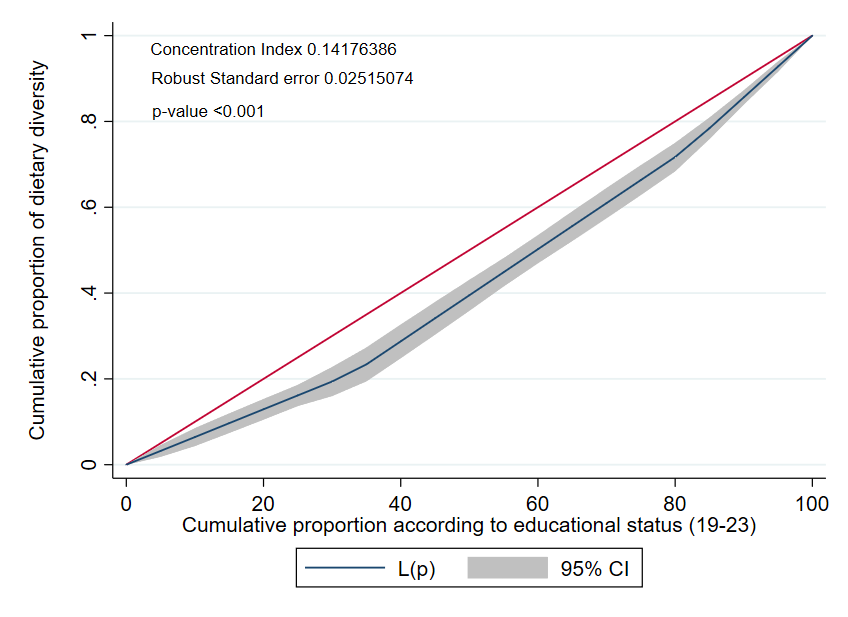


**Supplementary Fig. S22.** Concentration curve of dietary diversity among children aged 6-12 months against the wealth status of family.
